# Exaggerated Placental Site Reaction: A Challenging Diagnosis

**DOI:** 10.1155/crog/8854757

**Published:** 2026-04-27

**Authors:** Eleonora Nardi, Silvio Tartaglia, Davide Mulone, Ursula Catena, Antonio Lanzone, Vincenzo Arena

**Affiliations:** ^1^ Department of Laboratory and Hematological Sciences, Area of Pathology, Fondazione Policlinico Universitario Agostino Gemelli IRCCS, Rome, Italy; ^2^ Department of Woman′s and Child′s Health, Obstetrics Unit, Fondazione Policlinico Universitario Agostino Gemelli IRCCS, Rome, Italy; ^3^ Department of Diagnostics and Public Health, University of Verona, Verona, Italy, univr.it; ^4^ Department of Woman′s and Child′s Health, Gynecologic Oncology Unit, Fondazione Policlinico Universitario Agostino Gemelli IRCCS, Rome, Italy; ^5^ Section of Anatomic Pathology, Università Cattolica del Sacro Cuore, Rome, Italy, unicatt.it

**Keywords:** exaggerated placental site reaction, gestational trophoblastic diseases, intermediate trophoblast cells, pregnancy-related disorders

## Abstract

Gestational trophoblastic disease (GTD) includes both neoplastic and nonneoplastic disorders arising from placental trophoblastic tissue. Among tumor‐like conditions, exaggerated placental site reaction (EPSR) is a rare benign condition characterized by the persistence of the normal implantation site reaction following pregnancy. Histologically, it is characterized by diffuse infiltration of intermediate trophoblastic cells into the endometrium and superficial myometrium without features of malignancy. We report a case of EPSR presenting with persistent vaginal bleeding and rising *β*‐hCG levels after first‐trimester surgical termination of pregnancy, initially raising suspicion of retained products of conception and abnormal uterine vascularity on imaging. This case highlights the diagnostic challenges of EPSR and the importance of integrating clinical, biochemical, radiological, and histopathological findings to avoid overtreatment.

**Summary**

• Exaggerated placental site reaction (EPSR) is a rare, nonneoplastic complication of pregnancy.
• The awareness of this condition allows a multidisciplinary, safe, and successful management, reducing the risk of unnecessary and potentially harmful interventions.

## 1. Introduction

Gestational trophoblastic disease (GTD) encompasses a heterogeneous group of disorders arising from abnormal proliferation of placental trophoblastic tissue during or following pregnancy [1]. These conditions are broadly classified as benign or malignant. Benign forms include hydatidiform mole (complete and partial), whereas malignant gestational trophoblastic neoplasia (GTN) comprises invasive mole, choriocarcinoma, placental site trophoblastic tumor (PSTT), and epithelioid trophoblastic tumors [2]. In addition to these neoplastic conditions, a number of nonneoplastic trophoblastic lesions have been described, including placental site nodule (PSN) and EPSR [3].

EPSR is a benign condition characterized by diffuse infiltration of implantation‐site intermediate trophoblastic cells within the endometrium and superficial myometrium, representing an exaggerated form of the physiological implantation site reaction following pregnancy [3]. Typically, the normal structure of endometrial glands and myometrium is maintained [4]. Cases of EPSR are usually diagnosed following curettage for abortion or retained product of conception (RPOC), very often due to vaginal bleeding with abnormal uterine findings at pelvic ultrasound [4, 5]. Diagnosis of EPSR has also been described after normal pregnancies or in cases of abnormally invasive placentation [6, 7].

Although EPSR is not a neoplastic process, it may clinically and radiologically mimic RPOCs, GTD, GTN, or other vascular uterine abnormalities, thereby posing significant diagnostic challenges. Histological examination is crucial for the definitive diagnosis of EPSR, and it should be distinguished from other placental lesions such as PSTT, PSN, and choriocarcinoma.

Given its rarity and the potential overlap with more aggressive trophoblastic disorders, recognition of EPSR is important to avoid misdiagnosis and unnecessary invasive treatments. We report a case of EPSR following elective pregnancy termination, presenting with persistent vaginal bleeding and abnormal imaging findings, initially raising suspicion of trophoblastic disease.

## 2. Case Presentation

**Figure 1:**
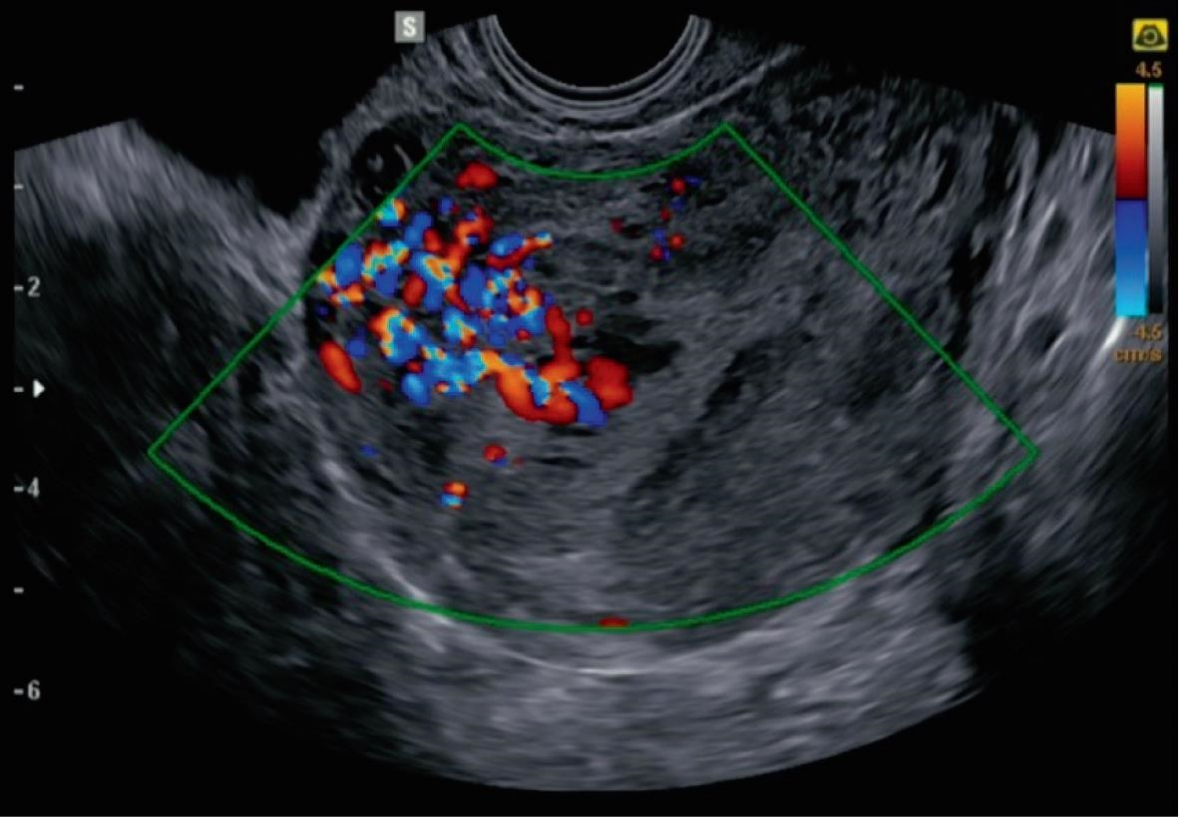
Transvaginal ultrasound revealing a heterogeneous endometrial mass with irregular borders and associated marked fundal myometrial hypervascularity on color Doppler.

A 35‐year‐old patient (G0P1) was referred to our unit for persistent vaginal bleeding 4 weeks after a voluntary termination of pregnancy (D&C) at 9 weeks of gestation. Serum *β*‐hCG levels were 234 mIU/mL 2 weeks after surgery and increased to 519 mIU/mL at 4 weeks. A transvaginal ultrasound revealed the presence of an endometrial heterogeneous, hyperechoic, vacuolated mass with irregular borders of 50 × 40 × 35 mm. Color Doppler imaging demonstrated increased vascular signals within the myometrium (Color Score 4) in the uterine fundal region (Figure 1). Spectral Doppler analysis revealed moderately high‐flow, low‐resistance waveforms, with slightly elevated peak systolic velocity (20 cm/s).

Given the documented, albeit infrequent, occurrence of arteriovenous malformation (AVM) and enhanced myometrial vascularization (EVM) following abortions [7] and the suspicious ultrasound findings, the patient underwent a preoperative uterine artery embolization to reduce the risk of massive bleeding.

**Figure 2:**
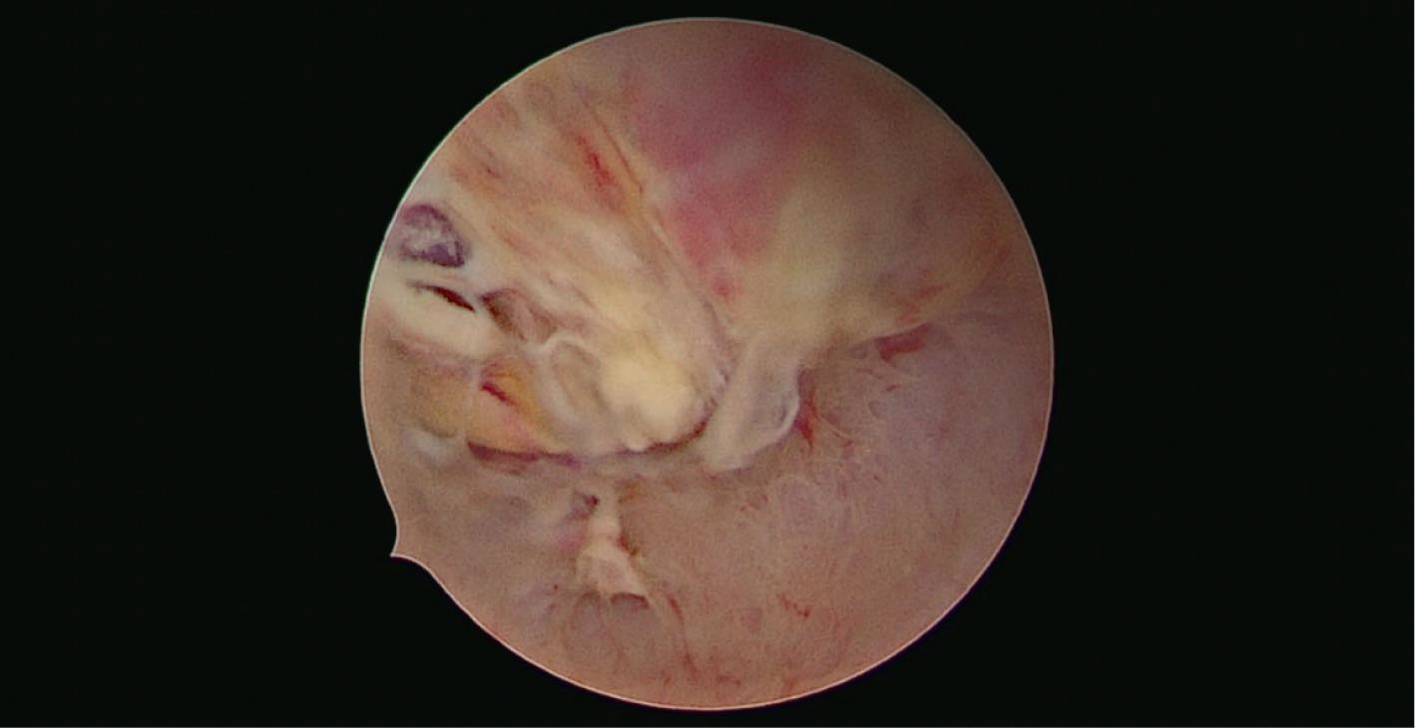
Hysteroscopic view of the irregular vascularized intrauterine mass adherent to the anterior wall of the uterus.

An ultrasound‐guided operative hysteroscopy was performed using a 6‐mm hysteroscope (TruClear Elite; Medtronic, Dublin, Ireland), revealing an irregular, friable, and highly vascularized intrauterine mass suggestive of RPOCs (Figure 2). The lesion appeared adherent to the anterior endometrial surface, with focal areas of contact bleeding. The mass was completely removed under direct visualization and sent to histological examination.

**Figure 3:**
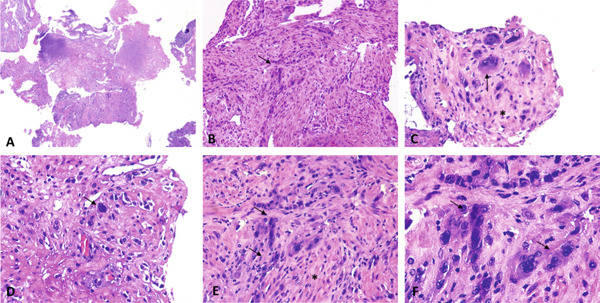
(A–F) H&E images showing mononucleate and multinucleate intermediate trophoblastic cells with eosinophilic cytoplasm and irregular nuclei (black arrow) invading the myometrium (asterisk): (A) magnification 2×, (B) magnification 10×, (C–E) magnification 20×, and (F) magnification 40×.

Upon gross examination, multiple grayish fragments measuring 4 × 4 cm were documented. Microscopic analysis revealed that the fragments consisted of residual placental parenchyma with fibrotic villi and involutive aspects interspersed with fibrin blood clots (Figure 3). Endometrial flaps, located near muscular elements, were observed with persistent intermediate trophoblast elements displaying modest atypia. These findings align with the characteristics of an exaggerated placental site. Vascular structures coexisted with endoluminal material, consistent with reported uterine artery embolization.

The postoperative course was uneventful, with no clinically significant vaginal bleeding. Serum *β*‐hCG levels declined appropriately and became undetectable within 2 weeks.

A follow‐up hysteroscopy performed 2 months later demonstrated a normal uterine cavity with no residual tissue.

## 3. Discussion

EPSR is a benign, nonneoplastic trophoblastic lesion that generally does not require aggressive treatment [8]. In most cases, management is conservative, consisting of clinical observation with serial monitoring of *β*‐hCG levels and ultrasound follow‐up, as the condition often resolves spontaneously. When EPSR presents with persistent vaginal bleeding or appears as an intrauterine mass mimicking RPOCs, hysteroscopic removal of the lesion may be performed to control symptoms and establish a definitive diagnosis while preserving fertility. More invasive procedures, such as hysterectomy, are rarely required and are typically reserved for cases complicated by severe hemorrhage or when the diagnosis remains uncertain, and a trophoblastic neoplasm cannot be excluded [9]. Chemotherapy is the main treatment for GTN. Low‐risk disease is usually managed with single‐agent regimens such as methotrexate or actinomycin D, while high‐risk cases require multiagent chemotherapy, most commonly the EMA‐CO regimen [2].

EPSR represents a diagnostic challenge for clinicians and has been relatively underrecognized in the literature.

Ultrasound plays a key role in evaluating suspected GTD and differentiating it from other trophoblastic or implantation‐site lesions. EPSR can be challenging to diagnose because its imaging features often resemble those of other trophoblastic conditions [10]. On transvaginal ultrasound, this nonneoplastic condition may appear as a heterogeneous intrauterine or endomyometrial lesion with variable vascularity, similar to RPOCs or, less frequently, GTN. Unlike GTN, however, the vascularity in EPSR is generally less disorganized and typically does not show the high‐velocity, low‐resistance flow patterns seen with arteriovenous shunting. Although RPOC may also present with endometrial thickening and increased Doppler signals, its vascular pattern usually stays confined within the endometrial cavity, not invading the myometrium [11]. Since these sonographic features are not definitive, preoperative diagnosis is challenging and depends on carefully integrating clinical history, serum *β*‐hCG trends, and histopathological analysis.

In the case presented, increasing serum *β*‐hCG, vaginal bleeding, and nonspecific ultrasound features represented potentially misleading signs. Direct visualization during the hysteroscopic procedure allows accurate, site‐specific sampling of trophoblastic tissue from the implantation area, but only histopathological examination allows a definitive, certain postoperative diagnosis.

The differential diagnosis between EPSR and other forms of GTD relies primarily on histopathological evaluation. EPSR is characterized by a diffuse infiltration of implantation site intermediate trophoblastic cells within the endometrium and superficial myometrium, typically arranged between myometrial fibers without significant cytologic atypia. Chorionic villi are usually present, and the proliferative activity is extremely low, with a Ki‐67 labeling index close to zero [12]. In contrast, PSTT demonstrates a more destructive and confluent myometrial infiltration by intermediate trophoblast, frequently associated with the absence of chorionic villi, increased cellular atypia, and a higher proliferative index [13]. Choriocarcinoma, another malignant form of GTN, is characterized by a biphasic proliferation of cytotrophoblast and syncytiotrophoblast, marked cytologic atypia, extensive hemorrhage and necrosis, and very high mitotic activity [14]. Therefore, the absence of significant atypia, preservation of chorionic villi, and the very low proliferative index are key histological features supporting the diagnosis of EPSR and distinguishing it from malignant trophoblastic neoplasms.

Uterine AVMs, including EMV, should also be considered in the differential diagnosis. These conditions may present with abnormal uterine vascularization and, in some cases, persistent or low‐level *β*‐hCG elevation, which may mimic EPSR and GTN. Microscopical evaluation of AVMs shows a mixture of abnormal and abnormally dilated blood vessels, including arteries, venules, and capillaries. In contrast to EPSR, AVM shows no signs of intermediate trophoblasts [15].

## 4. Conclusions

Clinicians should be aware of this possible reaction after uterine surgery for retained abortion or elective termination of pregnancy, especially in the first trimester of pregnancy. Due to the high risk of massive uterine bleeding, abnormal uterine lesions should be managed carefully by expert OB‐GYN surgeons to avoid possible severe complications. A conservative approach in cases of EPSR is feasible and may help avoid invasive and definitive procedures that can pose additional risks to the patient and, as in the case of hysterectomy, result in loss of future fertility.

In addition, more attention should be paid to trophoblastic pathology, highlighting the importance of histopathological examination. Further research is necessary to better understand these complications, for accurate differential diagnosis, detailed counseling of the patient, and safe surgical management.

## Author Contributions

Conceptualization: E.N. and V.A. Data acquisition: E.N., S.T., and U.C. Writing: E.N., S.T., and D.M. Review: S.T., U.C., A.L., and V.A. E.N. and S.T. contributed equally to this work.

## Funding

Open access publishing facilitated by Universita Cattolica del Sacro Cuore, as part of the Wiley ‐ CRUI‐CARE agreement.

## Ethics Statement

The present study complied with the Ethical Principles for Medical Research Involving Human Subjects, as outlined in the World Medical Association Declaration of Helsinki. Written informed consent was obtained from the patient for publication of this case report and any accompanying images. All clinical, instrumental, and histopathological data were fully anonymized prior to publication to ensure patient confidentiality.

## Conflicts of Interest

None of the authors have a conflict of interest to disclose.

## Supporting information


**Supporting Information** Additional supporting information can be found online in the Supporting Information section. The authors have completed the CARE checklist for case reports, which is available as Supporting Information.

## Data Availability

Data sharing not applicable to this article as no datasets were generated or analysed during the current study.
