# Supplementary material for: Exaggerated Placental Site Reaction: A Challenging Diagnosis
Source: Case Rep Obstet Gynecol. 2026 Apr 27;2026:8854757. doi: 10.1155/crog/8854757 (PMC13121087; doi:10.1155/crog/8854757)
Supplement: Supplementary file 1 — Supporting Information Additional supporting information can be found online in the Supporting Information section. The authors have completed the CARE checklist for case reports, which is available as Supporting Information. [file CROG-2026-8854757-s001.pdf]

CARE
case report guidelines

CARE Checklist of information to include when writing a case report

(cc) BY-NC-ND

Topic	Item	Checklist item description	Reported on Line
**Title**	1	The diagnosis or intervention of primary focus followed by the words "case report"	32
**Key Words**	2	2 to 5 key words that identify diagnoses or interventions in this case report, including "case report"	39-40
**Abstract (no references)**	3a	Introduction: What is unique about this case and what does it add to the scientific literature?	72-75
	3b	Main symptoms and/or important clinical findings	81-82
	3c	The main diagnoses, therapeutic interventions, and outcomes	107-110;121-122
	3d	Conclusion—What is the main "take-away" lesson(s) from this case?	186-189
**Introduction**	4	One or two paragraphs summarizing why this case is unique (**may include references**)	152-156
**Patient Information**	5a	De-identified patient specific information.	78
	5b	Primary concerns and symptoms of the patient.	78-79
	5c	Medical, family, and psycho-social history including relevant genetic information	78-79
	5d	Relevant past interventions with outcomes	79-80
**Clinical Findings**	6	Describe significant physical examination (PE) and important clinical findings.	78; 80-81
**Timeline**	7	Historical and current information from this episode of care organized as a timeline	78-81
**Diagnostic Assessment**	8a	Diagnostic testing (such as PE, laboratory testing, imaging, surveys).	80, 82, 84
	8b	Diagnostic challenges (such as access to testing, financial, or cultural)	91-93
	8c	Diagnosis (including other diagnoses considered)	110-111
	8d	Prognosis (such as staging in oncology) where applicable	N.A.
**Therapeutic Intervention**	9a	Types of therapeutic intervention (such as pharmacologic, surgical, preventive, self-care)	95
	9b	Administration of therapeutic intervention (such as dosage, strength, duration)	95-96
	9c	Changes in therapeutic intervention (with rationale)	N.A.
**Follow-up and Outcomes**	10a	Clinician and patient-assessed outcomes (if available)	119, 121-122
	10b	Important follow-up diagnostic and other test results	120
	10c	Intervention adherence and tolerability (How was this assessed?)	98-100
	10d	Adverse and unanticipated events	N.A.
**Discussion**	11a	A scientific discussion of the strengths AND limitations associated with this case report	137-138
	11b	Discussion of the relevant medical literature **with references**.	157-176
	11c	The scientific rationale for any conclusions (including assessment of possible causes)	179-182
	11d	The primary "take-away" lessons of this case report (without references) in a one paragraph conclusion	182-185
**Patient Perspective**	12	The patient should share their perspective in one to two paragraphs on the treatment(s) they received	N.A.
**Informed Consent**	13	Did the patient give informed consent? Please provide if requested	Yes ☒ No ☐
